# Efficacy of Simparica Trio® against induced infections of *Ancylostoma braziliense* and *Ancylostoma ceylanicum* in dogs

**DOI:** 10.1186/s13071-025-06758-3

**Published:** 2025-04-28

**Authors:** Raj Packianathan, Andrew Hodge, Natalie Bruellke, Michael Pearce, Frans Selepe, Piyanan Taweethavonsawat, Thomas Geurden

**Affiliations:** 1 Zoetis, Veterinary Medicine Research and Development, Zoetis Australia, Rhodes, NSW 2138 Australia; 2 Clinvet International, Uitzich Road, Bainsvlei, Bloemfontein, 9338 South Africa; 3 Parasitology unit, Faculty of Veterinary Science, Chulalongkorn University, Bangkok, Thailand; 4 Zoetis, Veterinary Medicine Research and Development, Mercuriusstraat 20, 1930 Zaventem, Belgium

**Keywords:** *Ancylostoma*, L_4_, L_5_, Moxidectin, Zoonosis

## Abstract

**Background:**

Hookworm infections such as *Ancylostoma braziliense* and *A. ceylanicum* pose a significant threat to pets and are implicated in causing zoonotic diseases. Despite the availability of preventatives, compliance can be lacking. Increasing pet owner options and combining endo- and ectoparasite treatments might improve this compliance. In four separate studies, we investigated the efficacy of Simparica Trio® (Zoetis Inc., Parsippany, NJ, USA) containing minimum dosages of 1.2 mg/kg sarolaner, 24 µg/kg moxidectin and 5 mg/kg pyrantel against fourth- and fifth-stage larvae (L_4_ and L_5_, respectively) and adults of *A. braziliense* and adults of *A*. *ceylanicum* in dogs.

**Methods:**

Four negatively controlled, randomised and blinded laboratory studies were conducted against induced infections of *A. braziliense* and *A. ceylanicum*, with the interval between infection and treatment selected to evaluate efficacy against each targeted stage. Each treated dog received a single oral dose of Simparica Trio® at the recommended label dose. Necropsy was conducted for worm recovery on day 7 or 8 post-treatment.

**Results:**

No treatment-related adverse events were recorded in any of the studies. No worms were recovered from any of the Simparica Trio®-treated dogs in all four studies, thus resulting in 100% efficacy (*P* ≤ 0.0005) of Simparica Trio® against all stages of *A. braziliense* and the adult stage of *A. ceylanicum* in dogs.

**Conclusions:**

Simparica Trio® containing sarolaner, moxidectin and pyrantel was highly effective against induced infections of L_4_, L_5_ and adult stages of *A. braziliense* and the adult stage of *A. ceylanicum* in dogs.

**Graphical Abstract:**

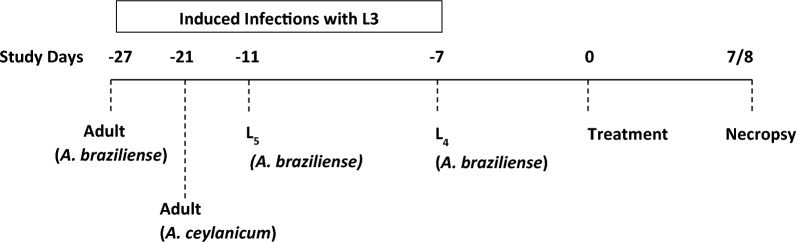

## Background

*Ancylostoma* spp. of hookworms are considered to be universal parasites, but their prevalence varies geographically depending on climatic conditions [1]. While *Ancylostoma caninum* and *Ancylostoma braziliense* have been reported worldwide, the prevalence of *A. ceylanicum* is predominantly confined to the Asia–Pacific region [2–17]. However, sporadic cases of *A. ceylanicum* have started to appear in Africa [18], South America [19–21], Central America [21] and Europe [22], highlighting the need for enhanced surveillance of its emergence in non-endemic countries [23]. *Ancylostoma braziliense* is endemic in subtropical regions [24] predominantly in Africa [18, 25–30], the Asia–Pacific [2], some parts of North America and South America [2, 31].

*Ancylostoma ceylanicum* causes blood loss and anaemia, particularly in young puppies [31], while *A. braziliense* only causes mild clinical signs in dogs [24]. In humans, however, *A. braziliense* is one of the most common causes of cutaneous larva migrans [2, 31–35], while *A. ceylanicum* can complete its life-cycle, leading to iron deficiency and anaemia, potentially leading to malnutrition in children and diarrhoea and eosinophilia in adults [5, 6, 14, 15]. Climate change, human mobility and urbanisation, which often result in co-habitation of humans and pets, will alter the epidemiology of infectious diseases, leading to an increase in zoonotic diseases [36–40].

Chemoprophylaxis has been a cornerstone of parasite control in dogs, and hookworms are no exception [41]. Despite the availability of various classes of monthly anthelmintics, such as macrocyclic lactones, tetrahydropyrimidines and benzimidazoles, owner compliance remains low due to lack of awareness regarding the significance of parasitic diseases in companion animals and their zoonotic potential [42]. Although anthelmintic resistance is rarely reported in dogs and cats [43], it is gradually emerging, with recent cases of multidrug resistant (MDR) *A. caninum* in the USA [44–52] and Canada [53] and pyrantel resistance in Australia [54–56]. The emergence of anthelmintic resistance, coupled with the morbidity caused by these worms worldwide, could have significant implications for both animal and human health [6].

We report here four studies that evaluated the efficacy of a single oral dose of Simparica Trio® (Zoetis Inc., Parsippany, NJ, USA) containing sarolaner, moxidectin and pyrantel against induced fourth- and fifth-stage larvae (L_4_ and L_5_, respectively) and adult *A. braziliense* and adult *A. ceylanicum* infections in dogs.

## Methods

Four negatively controlled, randomised and blinded laboratory studies were conducted to assess the efficacy of Simparica Trio® against induced infections. These studies focused on different stages: L_4_ stage (study 1), L_5_ stage (study 2) and adult stages of *A. braziliense* (study 3) and adult stages of *A. ceylanicum* infections (study 4). These studies were conducted in accordance with the World Association for the Advancement of Veterinary Parasitology (WAAVP) guidelines for evaluating the efficacy of anthelmintics for dogs and cats [57]; Veterinary International Cooperation on Harmonisation (VICH): Efficacy of anthelmintics: general requirements GL7 [58]; Efficacy of anthelmintics: specific recommendations for canines GL19 [59]; and Good Clinical Practice GL9 [60]. Each study was approved either by the Clinvet International Institutional Animal Care and Use Committee (studies 1–3) or Chulalongkorn University Animal Care and use Committee (study 4). Masked personnel performed clinical observations and parasitological evaluations in these studies.

### Animals

Purpose-bred laboratory Beagles and mixed breeds, males and females, that were confirmed to be clinically normal were enrolled in these studies. All dogs had undergone an adequate wash-out period of at least 21 days to ensure that no residual anthelmintic activity remained from any previously administered treatments. As a clean-out dewormer, only a commercial anthelmintic with activity that was short-acting and mainly limited to the gastrointestinal tract (e.g. pyrantel) was used. No macrocyclic lactones were administered.

All dogs were vaccinated according to the procedures at the study site but not within 1 week before or after inoculation nor within 1 week prior to dosing.

All dogs were acclimatised for at least 7 days prior to inoculation and were allowed to comingle until the treatment day (day 0). Dogs were housed in groups before treatment and individually housed after treatment. Commercial food rations were provided twice daily, and water was available ad libitum. The general health of all study dogs was monitored at least twice daily from acclimatisation until the end of the study.

### Experimental design

The puppies enrolled in the studies were aged between 9 and 15 weeks on the treatment day. All animals were confirmed to be free of gastrointestinal nematode infections prior to inoculation based on faecal examination using the McMaster chamber technique [61]. Each study consisted of two treatment groups: untreated controls (*n* = 8) and Simparica Trio®-treated (*n* = 8).

In each study, artificial inoculation of approximately 300 third-stage (L_3_), infective larvae was administered orally to each puppy, with *A. braziliense* larvae inoculated on day - 7 (study 1), day - 11 (study 2) or day - 27 (study 3) and *A. ceylanicum* larvae inoculated on day - 21 (study 4) [57, 61]. Viability was confirmed by the motility of the larvae, as observed using a microscope [62]. Prior to each artificial inoculation, food was withheld overnight and maropitant (Cerenia® [maropitant citrate]; Zoetis Inc.) was administered as an antiemetic at the recommended dose rate at least 1 h prior to inoculation, except in study 4. Each dog was observed for signs of vomiting after inoculation for up to 3 h post-inoculation and clinical observations were performed for up to 2 days post-inoculation.

### Induced infections

The *A. braziliense* isolates used in studies 1, 2 and 3 were originally collected from Port Alfred, Eastern Cape in the Republic of South Africa (RSA) in 2016. This RSA isolate was propagated 3 times in donor dogs, and larvae were collected and stored until used in these studies. In study 4, a Thailand isolate of *A. ceylanicum* was used, and challenge inoculum was prepared as previously described [61]; speciation was performed using quantitative PCR [63].

### Randomisation and treatment administration

Following the induced infections, dogs were randomly allocated to each treatment group according to a randomised complete block design, by blocking either on pre-treatment faecal egg counts (studies 3 and 4) or on pre-treatment body weights (studies 1 and 2). Following the induced infections, faecal egg counts were conducted in studies 3 and 4 to confirm that the challenge was successful. In study 3, the arithmetic mean pre-treatment faecal egg counts were 295.9 in the Simparica Trio®-treated group and 309.1 in the untreated group, whereas in study 4, mean counts were 7257 and 6794 in the Simparica Trio®-treated and untreated group, respectively. In both studies, pre-treatment egg counts were numerically similar.

On day 0, all study dogs were either treated with a single oral dose of Simparica Trio® or remained untreated. Each dog in the Simparica Trio®-treated group received a single oral dose of Simparica Trio® (actual dose administered: 1.33–2.22 mg/kg sarolaner, 27–44 µg/kg moxidectin and 5.56–9.26 mg/kg pyrantel [as pamoate salt]; Zoetis Inc.) according to the recommended label dose rate. Body weights recorded on day - 1 to 0 were used for dose selection. All dogs were monitored after dosing, and no evidence of expulsion of tablets was recorded. In study 4, one dog received an incorrect dose of Simparica Trio® and data from this dog was subsequently removed from the efficacy analysis (Table 1).

### Necropsy and worm counts

The euthanasia and necropsy carried out on day 7 or 8 followed a random order. After food was withheld at least 15 h prior to euthanasia, dogs were humanely euthanised with phenobarbital sodium following sedation with intramuscular acepromazine (study 1–3) or intravenous propofol (‘Lipuro’ [propofol]; B. Braun (Thailand) Ltd., Bangkok, Thailand) in study 4.

Following euthanasia, the entire gastrointestinal tract (from distal oesophagus to rectum) was removed from each dog, split longitudinally and the mucosal surface scraped twice with a suitable object, such as a microscope slide, to remove attached hookworms. For the L_4_ and L_5_ studies, the gastrointestinal contents and the scrapings were washed over a sieve with a mesh size of 38 µm. For the adult worm studies, the stomach contents and the small intestine scrapings were washed over a sieve with a mesh size of 150 µm, and the large intestine contents were washed over a sieve with a mesh size of 300 µm. For enumeration, worms were examined under a stereo microscope and counted; species were identified under the microscope based on morphology, developmental stage and sex (for L5 and adult worms) [62]. In each study, all detected stages of worms were counted and included in the efficacy analysis. No other helminth worms were found in any of these studies.

### Statistical analysis

The experimental unit was the pair of dogs. Using the PROC MIXED procedure (SAS 9.4; SAS Institute Inc., Cary, NC, USA), worm counts were natural log transformed (count + 1) prior to analysis. The log-counts of the untreated group (C) were compared to the log-counts of the Simparica Trio®-treated group (T) using a mixed linear model, with the fixed effect of treatment and the random effects of room (study 4 only), block, the interaction of block and treatment and error. Geometric least-squares (LS) mean counts were calculated from the LS mean for log-transformed counts and reported by treatment group. Percent reduction based on geometric LS mean was calculated using Abbott’s formula [(C-T)/C] × 100. Comparisons of mean counts (log scale) were conducted at the two-sided 0.05 level of significance between the untreated group and Simparica Trio®-treated group.

## Results

No treatment-related adverse events were recorded in any of the studies following treatment. Episodes of mild diarrhoea were recorded in some dogs prior to treatment in both groups due to the induced infections in studies 1–3. All animals in the untreated control groups had adequate infestations and complied with the 'Efficacy of anthelmintics: specific recommendations for canines GL19 [59] and WAAVP guidelines [57]. Efficacy results are summarised in Table 1.

**Table 1:** Efficacy of a single dose of Simparica Trio® containing sarolaner, moxidectin and pyrantel pamoate against induced infections of larval (L_4_), immature adult (L_5_) and adult stages of *Ancylostoma braziliense* and the adult stage of *A. ceylanicum* in dogs

Study	Parasite—origin	Stage at time of treatment	Day of inoculation	Day of worm counts	Treatment group^a^	*N*	Sex of dog	Mean age of dogs (weeks)^b^	Mean body weight of dogs (kg)^c^	Number of infected dogs	Total worm counts		Efficacy (%)	Efficacy compared to controls	
											Range (*n*)	AM (GM)		*P* value	Test statistic
1	*A. braziliense*—RSA	L_4_ larvae	− 7	8	Untreated	8	2F, 6M	9.8	3.46	8	134–211	173.1 (171.2)	–	–	–
					Simparica Trio®	8	4F, 4M	9.8	3.43	0	0–0	0.0 (0.0)	100	< 0.0001	*t*_(3)_ = 90.72
2	*A. braziliense*—RSA	Immature adult (L_5_)	− 11	8	Untreated	8	2F, 6M	12.0	3.55	8	115–198	163.6 (160.7)	–	–	–
					Simparica Trio®	8	5F, 3M	12.0	3.39	0	0–0	0.0 (0.0)	100	< 0.0001	*t*_(3)_ = 67.20
3	*A. braziliense*—RSA	Adult	− 27	7	Untreated	8	4F, 4M	14.6	7.40	8	215–380	295.8 (291.3)	–	–	–
					Simparica Trio®	8	4F, 4M	14.5	7.43	0	0–0	0.0 (0.0)	100	< 0.0001	*t*_(3)_ = 83.07
4	*A. ceylanicum*—Thailand	Adult	− 21	7	Untreated	8	6F, 2M	15.0	6.65	8	60–271	139.0 (121.6)	–	–	–
					Simparica Trio®	7	2F, 5M	15.0	6.70	0	0–0	0.0 (0.0)	100	0.0005	*t*_(3)_ = 16.91

*AM* Arithmetic mean,* F* female,* GM* geometric mean,* L4* fourth-stage larvae,* L5* fifth-stage larvae,* M* male,* N* number of animals per group,* RSA* Republic of South Africa

^a^Simparica trio® provided a minimum of 1.2 mg/kg sarolaner, 24 µg/kg moxidectin and 5 mg/kg pyrantel

^b^Age on day 0 when treatment was administered

^c^Body weights were recorded on day - 1 (studies 1–3) or day 0 (study 4)

### *Ancylostoma braziliense*

Worms were recovered from all untreated dogs in studies 1–3. Geometric mean worm counts in the untreated dogs were 171.2 (range 134–211) in study 1, 160.7 (range 115–198) in study 2 and 291.3 (range 215–380) in study 3. In all three studies, geometric mean worm counts were significantly (test statistic *t*_(3)_ = 67.20–90.72;* P* < 0.0001) higher in the untreated group whereas no worms were recovered in the Simparica Trio®-treated group, resulting in an efficacy of 100% based on assessments using both arithmetic and geometric means against L_4_ and L_5_ and adult stages of *A. braziliense*.

### *Ancylostoma ceylanicum*

In study 4, 60–271 worms were recovered from all untreated dogs. The geometric mean worm count in the untreated dogs was 121.6 whereas no worms were recovered in the Simparica Trio®-treated group. The geometric mean worm count was significantly (*t*_(3)_ = 16.91;* P* = 0.0005) lower in the Simparica Trio®-treated group than in the control group. Overall efficacy of Simparica Trio® was 100% against the adult stages of *A. ceylanicum*.

## Discussion

Hookworm infections pose a significant threat to dogs worldwide [1]. Despite the availability of numerous preventive treatments, both over the counter and through veterinary channels, pet owner compliance with regular monthly treatment is still low [42]. Since both *A. braziliense* and *A. ceylanicum* can cause zoonotic diseases and given the rapid rise of *A. ceylanicum* in both endemic and non-endemic regions [2, 3], the education of pet owners by veterinarians about the zoonotic potential of these worms is critical to complementing WHO’s One Health roadmap for eliminating hookworms by the 2030s [14, 64].

Recent reports of anthelmintic resistance in companion animals are alarming, particularly resistance against hookworms [43]. Although most of these reports originate from the USA and Canada with MRD *A. caninum*, including resistance to pyrantel, benzimidazole and milbemycin oxime [44–52, 54], pyrantel resistance has also been reported in Australia [54–56]. More studies are required to investigate similar issues with other hookworms, such as *A. braziliense* and *A. ceylanicum* [6]. Using a combination of different classes of anthelmintics has long been regarded as the best strategy to combat and delay the onset of anthelmintic resistance [65]. Combinations of different classes of anthelmintics, such as combinations of moxidectin, emodepside, pyrantel, and febantel, have proven to be effective against MDR *A. caninum* in dogs [46, 48, 66].

Simparica Trio®, which contains two different classes of anthelmintics, namely moxidectin and pyrantel, has been shown to be highly effective in the control of various roundworms and hookworms in dogs in the USA and Europe [62, 67, 68]. Simparica Trio® was designed to contain a higher concentration of moxidectin, with the aim to enhance efficacy against resistant heartworms [69] as the standard dose has been shown to fail [70]. Similar benefits of moxidectin against MDR hookworms need to be investigated.

In the studies reported here, Simparica Trio® provided 100% efficacy against all three stages of *A. braziliense*, as reported in previous studies against *A. caninum* [62]. Milbemycin oxime in combination with afoxolaner at the recommended label dose provided 90.0% efficacy against an induced infection with the USA isolates of *A. braziliense* in a laboratory study [71] and 94.8–98.0% efficacy against natural infections of *A. braziliense* in the RSA [27, 30], and provided 99.9% efficacy against induced infection of *A*. c*eylanicum* in dogs [72]. Moxidectin efficacy against *A. ceylanicum* has previously been reported [61].

## Conclusions

A single oral dose of Simparica Trio® containing sarolaner, moxidectin and pyrantel was highly effective against induced infections of L_4_, L_5_ and adult stages of *A. braziliense* and the adult stage of *A. ceylanicum* in dogs.

## Data Availability

Relevant datasets generated and/or analysed during these studies are included within the article.

## Abbreviations

MDR: Multidrug resistant
RSA: Republic of South Africa
